# Study on the migration characteristics of bioaerosols and optimization of ventilation patterns in a negative pressure isolation ward considering different patient postures

**DOI:** 10.1371/journal.pone.0290288

**Published:** 2023-08-17

**Authors:** Dieen Wu, Jianji Chen, Xihua Fu, Zongkun Li, Futai Tan, Hai Lin

**Affiliations:** 1 Guangzhou Panyu Central Hospital, Guangzhou, China; 2 School of Electric Power, South China University of Technology, Guangzhou, China; 3 Guangdong Province Key Laboratory of Efficient and Clean Energy Utilization, Guangzhou, China; 4 Guangzhou Huijin Energy Efficiency Technology Co. Ltd, Guangzhou, China; Macquarie University, AUSTRALIA

## Abstract

Due to the serious global harm caused by the outbreak of various viral infectious diseases, how to improve indoor air quality and contain the spread of infectious bioaerosols has become a popular research subject. Negative pressure isolation ward is a key place to prevent the spread of aerosol particles. However, there is still limited knowledge available regarding airflow patterns and bioaerosol diffusion behavior in the ward, which is not conducive to reducing the risk of cross-infection between health care workers (HCWs) and patients. In addition, ventilation layout and patient posture have important effects on aerosol distribution. In this study, the spatial and temporal characteristics as well as dispersion patterns of bioaerosols under different ventilation patterns in the ward were investigated using the computational fluid dynamics (CFD) technique. It is concluded that changes in the location of droplet release source due to different body positions of the patient have a significant effect on the bioaerosol distribution. After optimizing the layout arrangements of exhaust air, the aerosol concentration in the ward with the patient in both supine and sitting positions is significantly reduced with particle removal efficiencies exceeding 95%, that is, the ventilation performance is improved. Meanwhile, the proportion of aerosol deposition on all surfaces of the ward is decreased, especially the deposition on both the patient’s body and the bed is less than 1%, implying that the risk of HCWs being infected through direct contact is reduced.

## 1. Introduction

In recent decades, all parts of the world have been hit by some large-scale viral infections, which not only hinders the economic development of countries around the world, but also seriously threatens human life and health [**1**]. The sharp increase in the need to treat patients with that has put enormous pressure on hospital operations and the allocation of medical resources. Epidemiological studies have shown that the main infection routes of virus are airborne and direct contact transmission, which means that it has a strong transmission ability [2, 3]. In order to give patients effective treatment, many hospitals have built negative pressure isolation wards. The air pressure in the isolation ward is lower than the adjacent space to prevent contaminated air from spreading to the outside environment [4, 5]. In the ward, the supply air flow rate is less than the exhaust air flow rate, resulting in negative pressure [6]. Additionally, a buffer room is generally set between the isolation ward and the corridor to prevent the leakage of contaminated air during the door opening process. The construction of negative pressure isolation ward is crucial to curb the spread of the epidemic, in which pressurization, ventilation rate and filtration are the key factors to maintain the negative pressure isolation wards. Many countries have issued guidelines for the construction of negative pressure isolation rooms for their own countries, but there is still no unified design standard internationally. In this case, the requirements of isolation wards in China should be that the pressure difference with the adjacent room is not less than 5 Pa, and the number of air changes is between 10–15 ACH [7].

Due to the small particle size (mostly less than 5μm), droplet aerosols have the properties of following airflow streamlines, being suspended in the air, and having the potential to spread over long distances, which significantly increases the probability of cross-infection between health care workers (HCWs) and patients [8]. In infectious disease outbreaks, a large number of HCWs were infected. Therefore, an effective ventilation strategy is especially important to reduce cross-infection. However, because of the complexity of airflow movement, there is still insufficient research on key parameters such as vent location, number of air changes as well as air speed.

The diffusion of bioaerosol particles is mainly dependent on airflow. Computational fluid dynamics (CFD) simulations act as tools to simulate indoor airflow distribution and particle motion trajectory, so as to obtain the diffusion distribution law of particles. Hence, it is often used to design a better layout for supply and exhaust air to improve ventilation performance and achieve pollution control. Based on CFD simulation analysis, scholars have conducted some studies on the airflow pattern and pollutant distribution in the negative pressure isolation ward [9–11]. Cho et al. [12] and Hang et al. [13] used tracer gas, such as SF_6_, to study the spread of pathogens in an isolation room under different ventilation strategies. They found that the locations of inlet and outlet air streams are important factors affecting the pollutant removal efficiency. By optimizing ventilation strategies, the risk of cross-contamination in isolation rooms can be reduced. The ventilation arrangement was studied by Lu et al. [14]. Numerical calculation results show that the concentration of pollutants in the breathing area of HCWs under stratified ventilation is the lowest, thereby reducing the risk of infection. Using CO2 as a tracer gas, Wang et al. [15, 16] assessed the effect of the location of the exhaust vent on the ventilation effect. The results show that the arrangement of the exhaust vent near the head of the bed is beneficial to enhance ventilation performance. They proposed an air jet curtain for extra protection for HCWs. Since the tracer gas cannot reflect the size distribution and behavioral trajectory of aerosol particles, more studies have utilized the discrete phase model (DPM) to obtain more accurate pollutant diffusion laws. Lu et al. [17] revealed the distribution characteristics of cough droplets under stratified ventilation, mixed ventilation and displacement ventilation. They found by calculation that the stratified ventilation is better for the removal of droplets with a diameter of 50 μm, and the higher airflow speed can promote the deposition of droplets. Dao et al. [18] evaluated various air outlet arrangements and proposed the optimal exhaust vent location to maximize droplet removal efficiency in hospital isolation wards. Ren et al. [19] analyzed the motion trajectories of particles with different diameters using the Euler-Lagrangian model, and found that in different ventilation strategies, most of the large particles were deposited on solid surfaces in different areas of the ward. In summary, most researches focus on bioaerosol removal efficiency and ward ventilation performance when the patient is in a single position [11, 20]. However, in reality, the patient is not confined to a single position within the ward. The spatiotemporal distribution and deposition of bioaerosols released by the patient in different positions vary significantly. Moreover, the impact of different ventilation arrangements on these factors is not yet clear. The lack of adequate discussion of these issues in previous studies is not conducive to improving ward ventilation performance and reducing the risk of cross-infection.

To address the above issues, in this study, the airflow field and bioaerosol distribution were simulated using the Euler-Lagrange model with a single negative pressure isolation ward in a hospital. The migration characteristic of bioaerosols was quantitatively analyzed, and the spatiotemporal distribution of aerosol particles was predicted. Considering the interactive effects of ventilation layout and patient posture on ventilation performance, the ventilation layout of the ward was optimized to enhance pollutant control. Improved ventilation design for negative pressure isolation wards, thereby reducing the risk of cross-infection within the hospital.

## 2. Materials and methods

2.1 Case study

**Fig 1:**
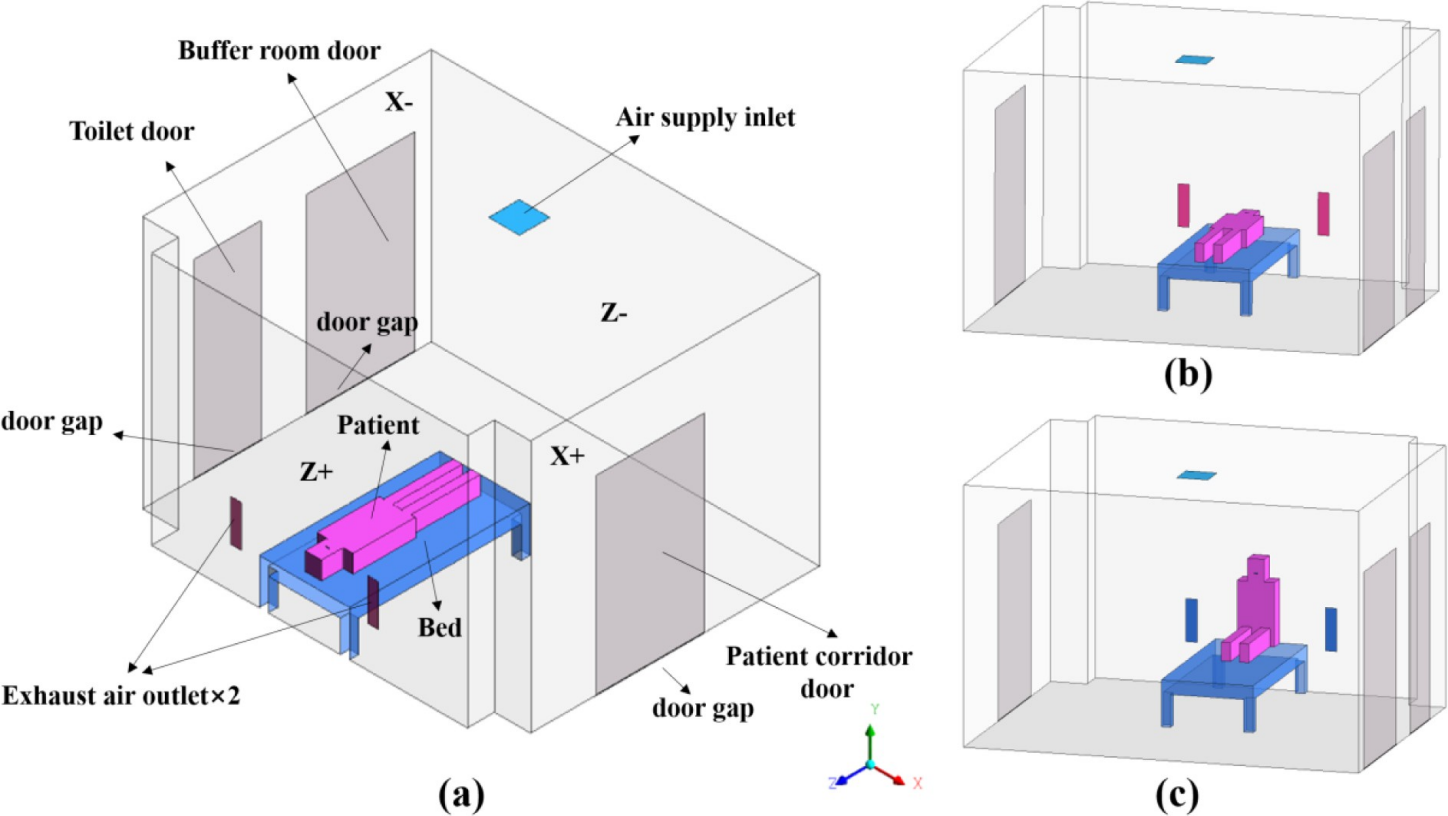
Physical model of the negative pressure isolation ward: (a) schematic layout of the ward; (b) with a patient in the supine position; (c) with a patient in the sitting position.

**Fig 2:**
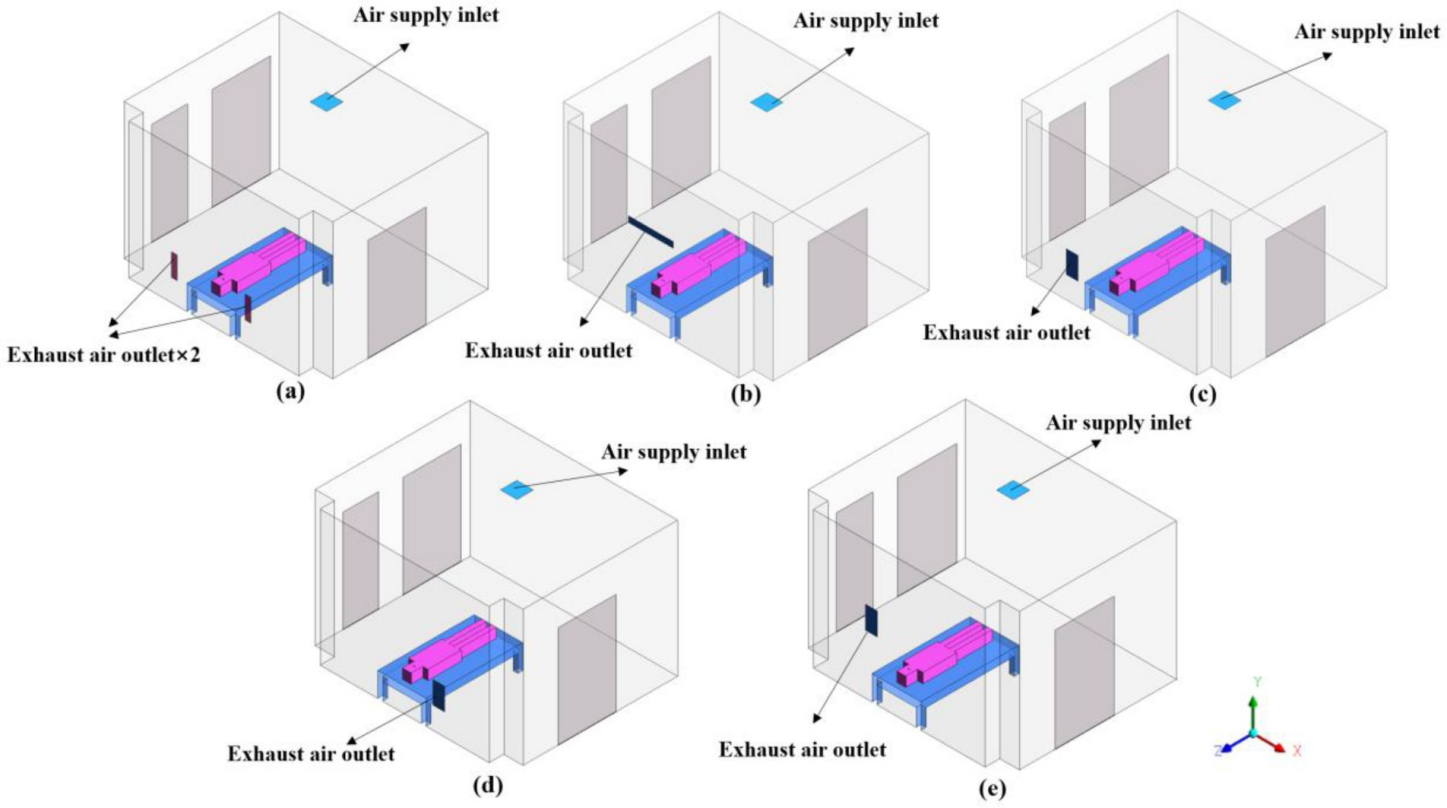
Layout of isolated wards based on ventilation strategies: (a)case1; (b)case2; (c)case3; (d)case4; (e)case5.

In this paper, a single negative pressure isolation ward in a hospital in Guangzhou is modeled and simulated. The dimensions of the ward are 4.3m length (L) × 3.5m width (W) × 2.8m height (H), as shown in Fig 1(A). A hospital bed is placed in the middle of the ward, and the patient on it is simplified as a block model. Three doors are installed in the ward, leading to the buffer room, the toilet and the patient corridor respectively, and there are 5mm gaps at the bottom of the doors. Except the ceiling and floor, the walls surrounding the interior of the ward are denoted by Z-, Z+, X-, and X+, respectively. Due to the good air-tightness of enclosure structure such as wall panels, the infiltration air volume only enters the ward from the door gap. In this study, five cases were analyzed based on the location of exhaust outlet to find out the best arrangement. As shown in Fig 1(B) and 1(C), two typical patient positions, supine(sup) and sitting(sit), were considered for each arrangement based on the influence of different release sources on droplet diffusion, and a total of ten cases were simulated (5×2). Various situations are described below according to the position of exhaust outlets. Fig 2 shows the different vent layouts in the isolation ward.

- Cases 1(sup&sit): Ceiling air supply passes through the patient, and two exhaust air outlets are located on both sides of the bed at a distance of 450mm from the floor.
- Cases 2(sup&sit): Considering the height of the patient sitting on the bed, the exhaust air outlet is installed above the patient at 1630mm from the floor.
- Cases 3(sup&sit): One exhaust air outlet is installed on the left side of the bed.
- Cases 4(sup&sit): One exhaust air outlet is located on the right side of the bed, opposite to case 3.
- Cases 5(sup&sit): The air outlet is arranged on the left side of the bed at 1200mm above the floor.

The sizes of the air supply inlets and exhaust air outlets for all cases are shown in Table 1.

**Table 1:** Sizes of the air supply inlets and exhaust air outlets.

Type	Cases	Size (L×W)
air supply inlet	Total	340mm×340mm
exhaust air outlet	1	460mm×120mm
	2	920mm×120mm
	3–5	460mm×240mm

### 2.2 Mathematical model and verification

In this study, the Euler-Lagrangian method is used to simulate the airflow field and bioaerosol distribution [21]. Because indoor airflow is generally turbulent, the Reynolds Averaged Navier-Stokes (RANS) model is often used for calculation [22]. The Realizable k-ε model is adopted as the turbulence model as it performs well in room ventilation [23, 24]. For the near wall, the standard wall function is used for processing. Since the volume ratio of aerosol particles is much less than 10%, the droplet model in DPM is used to simulate the diffusion behavior of particles [25]. Meanwhile, a discrete random walk (DRW) model is applied to predict the changes of particle motion trajectories caused by turbulence [26].

The governing equation for the airflow phase is shown below:

$$\frac{\partial (\rho \phi )}{\partial t}+\nabla •(\rho \phi V)=\nabla •(\Gamma_{\phi }\nabla_{\phi })+S_{\phi }$$ (1)

In Eq (1), ρ is the air density, *V* are the air velocity vectors. φ is the transport quantity, which can indicate the three-dimensional velocity component, temperature, or other physical quantities. Γ_*ϕ*_, S_*ϕ*_ represent the effective diffusion coefficient and the source term, respectively.

The force equation for bioaerosol particles can be expressed by the following formula:

$$\frac{{\mathrm{du}}_{p}}{dt}=F_{D}(u_{g}-u_{p})+\frac{g(\rho_{p}-\rho_{g})}{\rho_{p}}+\frac{F_{i}}{m_{p}}$$ (2)

Where, u_p_ and u_g_ are the velocity of particles and airflow, respectively. ρ_p_ and ρ_g_ are the density of particles and airflow, respectively. F_D_ represents drag. F_i_ is the additional force acting on the particles.

Since the airflow field and bioaerosol distribution in the ward are calculated using the Realizable k-ε model and the DPM model, respectively, the accuracy of these two models is critical to the accuracy of the overall calculation results. Therefore, these two models need to be experimentally validated.

**Fig 3:**
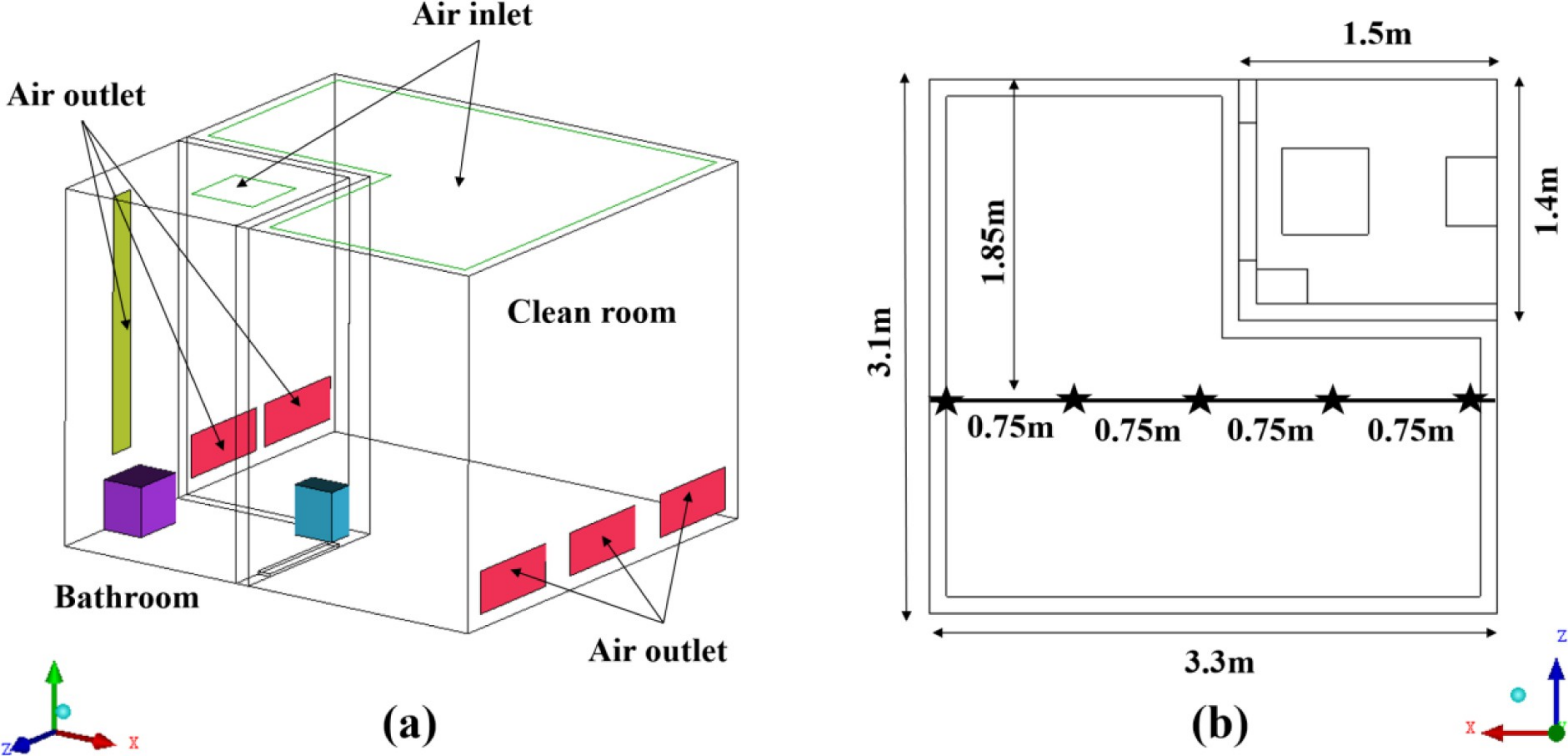
(a) The layout of the clean room and adjacent bathroom, (b) The location of air velocity measurement points.

**Fig 4:**
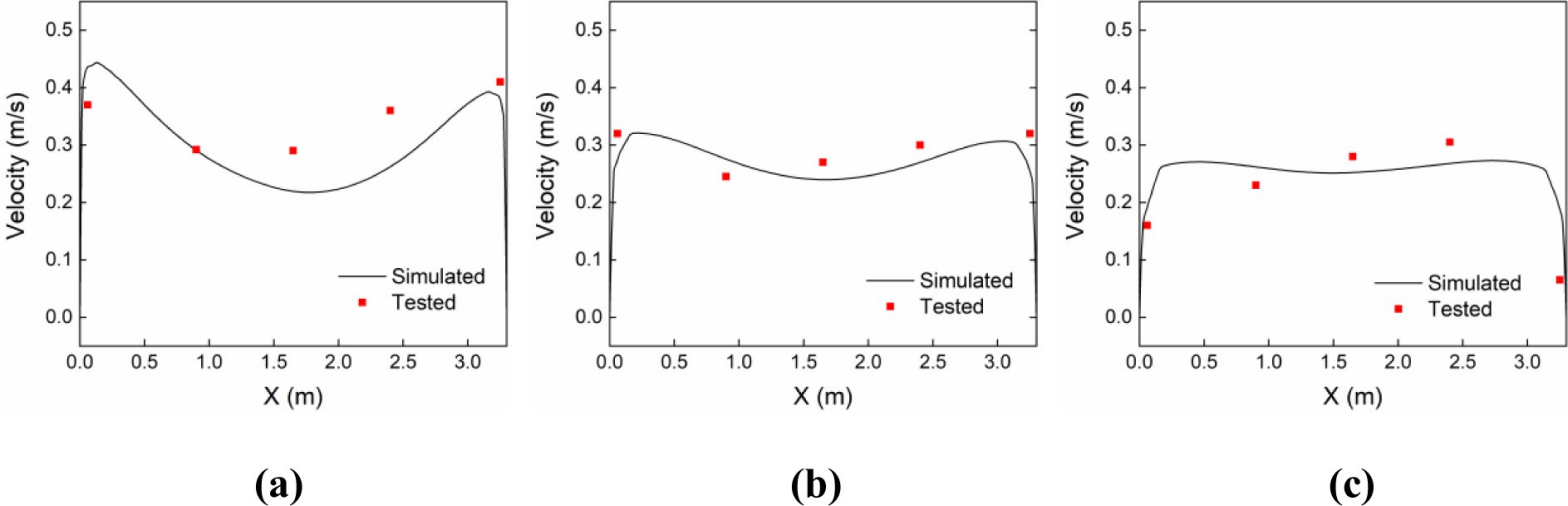
Simulated and measured air velocity data at three heights within the clean room: (a) 0.8 m, (b) 1.2 m, (c) 1.6 m.

To verify the correctness of the turbulence model, the numerical simulation results were compared with the airflow distribution data measured in the clean room by Yang et al. The layout of the clean room and the adjacent bathroom is shown in Fig 3(A). The clean room measured 3.3m (L) × 2.5m (H) × 3.1m (W), whereas the bathroom measured 1.5m (L) × 2.5m (H) × 1.4m (W). The air supply volume of the clean room was 6950 m^3^/h and the air exhaust volume was 6850 m^3^/h. After the clean air entered the clean room, part of it was discharged from the five air outlets on the side wall, and the other part entered the bathroom through the door gap. Additionally, the bathroom’s inlet and exhaust volumes were 325 m^3^/h and 425 m^3^/h, respectively. The locations of the air velocity measurement points, which were set up at three heights of 0.8 m, 1.2 m, and 1.6 m, respectively, are depicted in Fig 3(B). In this research, Realizable k-ε was used to simulate the airflow phase distribution and compared with the measured data, as shown in Fig 4. The findings indicated that the air velocity simulated values were in good agreement with the measured air velocities at each measurement point. Thus, it is feasible to replicate the flow field of the isolation ward using the Realizable k-model.

**Fig 5:**
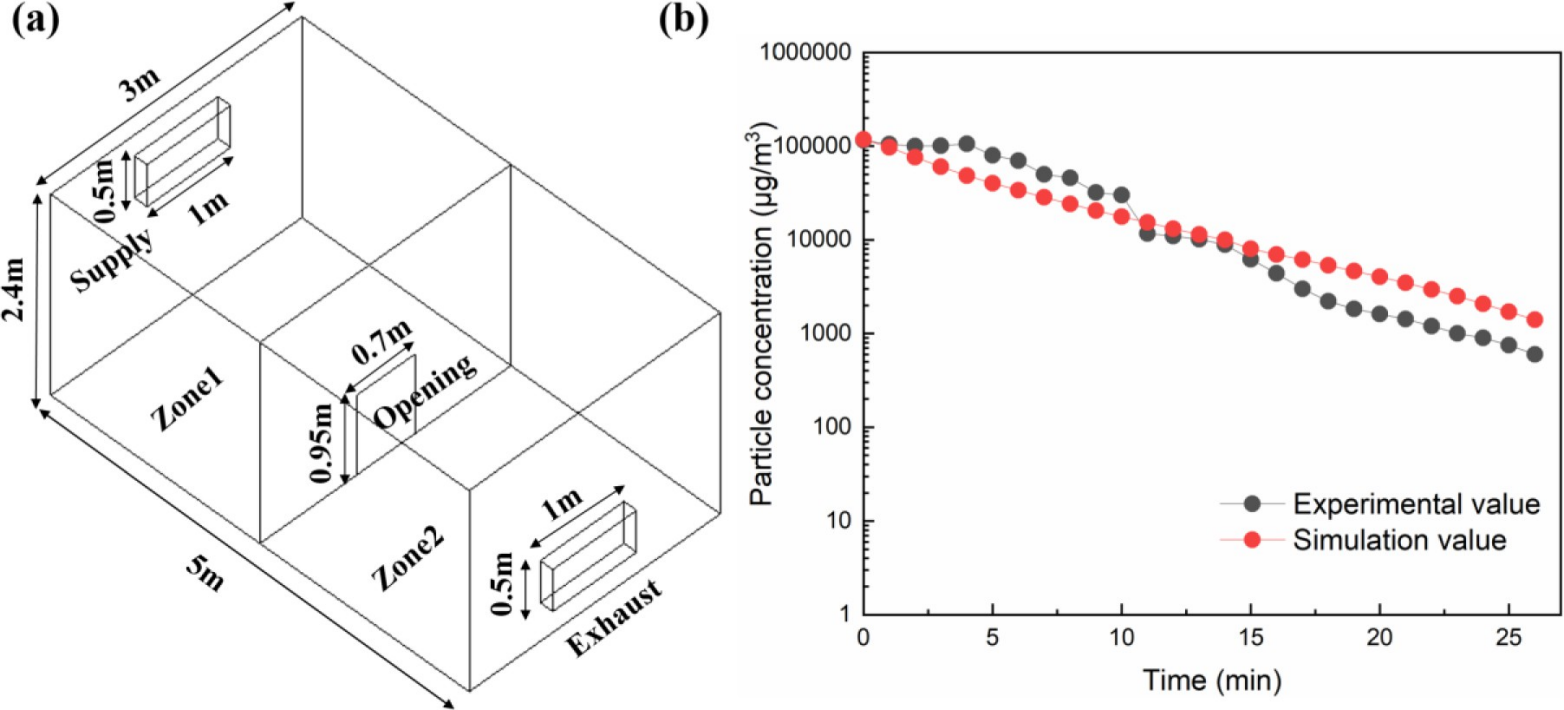
**(a)** Schematic diagram of the room; **(b)** Comparison of simulated and measured values of particle concentration with time.

The motion of discrete particles is calculated using the Euler-Lagrangian model. The accuracy of particle motion diffusion plays a crucial role in the correctness of the overall calculation results. And, the particle motion diffusion is realized by the Eulerian-Lagrangian model calculation. Therefore, verifying the reliability of this model is crucial to ensure the accuracy of particle motion diffusion. In order to achieve this, the experimental data of Lu et al. [27] are compared with our simulation results under the same conditions. Fig 5(A) displays the full-scale physical model of the experimental room with a geometric size of 5m(L)×2.4m(H)×3m(W). The air change rate inside the room was 9.2 ACH. The size ranges of the smoke particles were from 1.0 μm to 5.0 μm. The density of particles is 865.0 kg/m^3^. In the initial stage, these particles are uniformly arranged in zone 1 according to the specific conditions of the experiment, and the velocity is zero. After the experiment started, ventilation and openings were turned on. The airflow entered the room through the supply vents, directed the particles through the openings into Zone 2, and then exited through the exhaust vents. During this process, the particle concentration in Zone 1 was measured for 27 minutes. The comparison between the experimental data and the simulated data is shown in Fig 5(B). It can be seen that they are in good agreement and have the same trend of change, thus verifying the accuracy of the model used.

### 2.3 Boundary conditions

Fig 1(A) shows the arrangement of the buffer room door, toilet door, and patient corridor door. Considering the better airtightness of the doors in the actual situation, only the door gaps were provided at the bottom. Due to the good airtightness, the air leakage in the ward only occurred at the crack of the door. According to the requirements for pressure gradients in the Requirements of Environment Control for Hospital Negative Pressure Isolation Ward (GB/T 35428–2017) [7], the ward was -15 Pa, the buffer room was -10 Pa, the toilet was -20 Pa, and the patient corridor was -10 Pa. After measurement, the air velocity (temperature) at the door gap between the ward and buffer room is 1.8m/s (23°C), while the air velocity (temperature) at the toilet door gap and the patient corridor door gap are 1.7m/s (22°C) and 1.8m/s (23°C), respectively. In accordance with the design requirements of GB/T 35428–2017, the ventilation rate of the ward was 12ACH. Therefore, the air supply volume and air supply speed were 500m^3^/h and 1.2m/s, respectively. The supply air temperature and relative humidity were 21°C and 60%, respectively. The exhaust air outlet was set as a -15 Pa pressure outlet to maintain negative pressure in the ward. The patient’s body temperature was 34°C [28]. Because the ward is located inside the hospital, the heat load on the walls, ceiling and floor around the ward is small, so it can be set as a thermally insulated wall [29].

In this study, transient simulations were implemented to investigate the diffusion law of bioaerosols for a total simulation time of 500 s. According to Wang et al. [28], the release rate of bioaerosol particles is 214/s, which gives a total number of particle releases for the entire simulation of 107,000. They also noted that the diameter and initial velocity of the particles were 1 μm and 0.5 m/s, respectively. DPM droplets are injected into the space as the patient exhales and disperse rapidly. The droplet particles are composed of water, salt and glycerin, and their density, temperature and moisture content are 1028.9kg/m^3^, 37°Cand 92% respectively [30]. Meanwhile, the parameters of the patient’s exhaled airflow were set according to literature [31]. For isolation ward inlet and outlet, the boundary condition for droplets in DPM was set as escape condition. The trap condition in DPM was applied to the droplets at patient body and walls, assuming that the trajectory calculation terminates when they touch these surfaces. The ventilation system was fully evaluated by monitoring the accumulated aerosol concentration in isolation room. The specific boundary conditions are shown in Table 2.

**Table 2:** Boundary condition.

Parameter	Type	Value
Air supply inlet	Velocity inlet Discrete phase: escape	Velocity: 1.2m/s Temperature: 21°C Relative humidity: 60%
Exhaust air outlet	Pressure outlet Discrete phase: escape	Pressure: -15Pa
Buffer room door gap	Velocity inlet	Velocity: 1.8m/s Temperature: 23°C
Toilet door gap	Velocity inlet	Velocity: -1.7m/s Temperature: 22°C
Patient corridor door gap	Velocity inlet	Velocity: 1.8m/s Temperature: 23°C
Walls, door, floor, ceiling and bed	Wall Discrete phase: trap	Adiabatic
Exhaled air by patient	Velocity inlet	Velocity: 0.5m/s Temperature: 36°C Relative humidity: 90%
Bioaerosol	DPM: Injection	Velocity: 0.5m/s Diameter: 1μm Quantity: 214/s Moisture content: 92%
Patient	Wall	Temperature: 34°C

### 2.4 Mesh-independent verification

**Fig 6:**
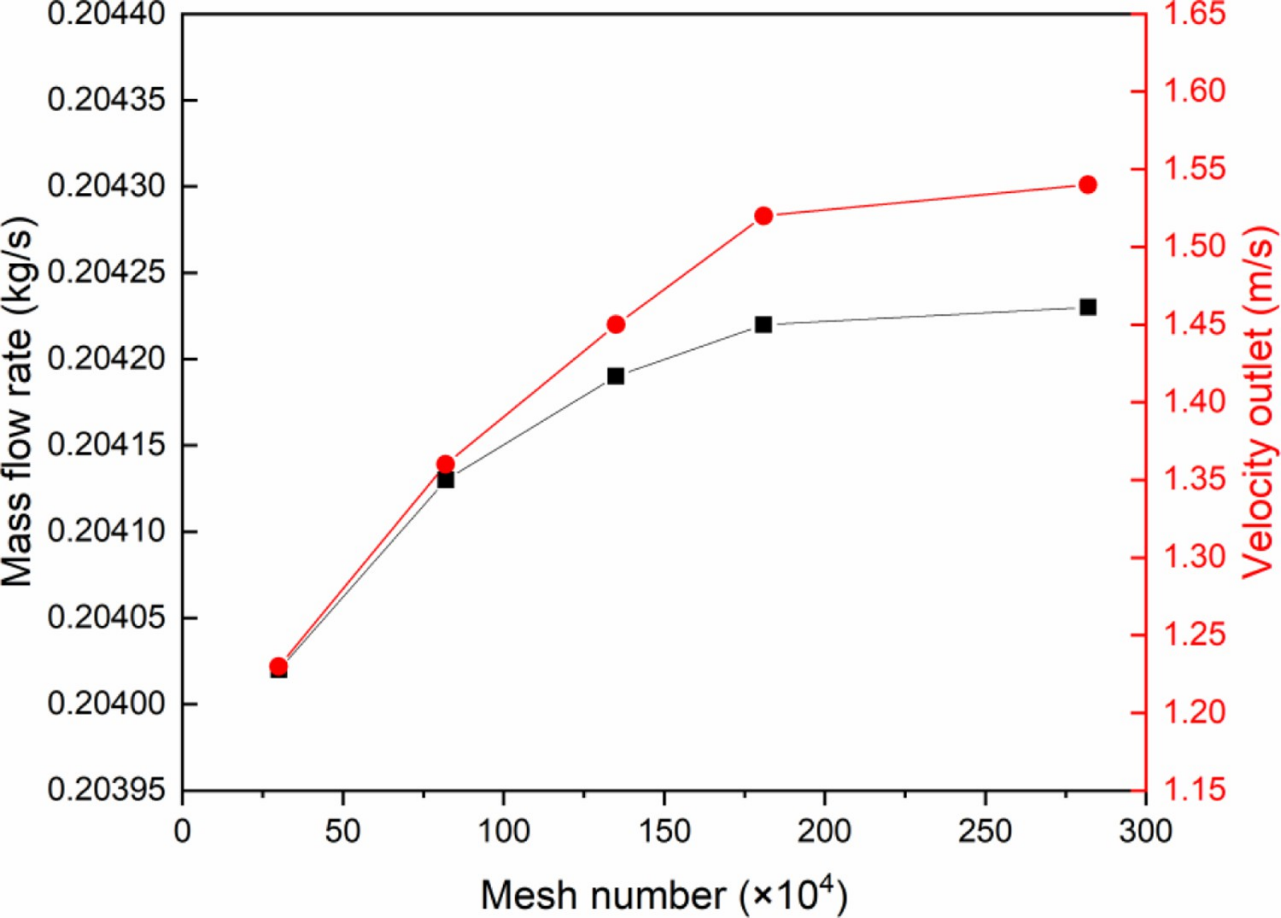
Mesh independent analysis based on mass flow rate and velocity outlet.

The finite control volume method divides the computational area into small cells with a tetrahedral mesh. Because of the large velocity or temperature gradients in areas such as air vents, human bodies, and door gaps, the mesh refinement method is used to improve the accuracy of calculation results. In the process of refining mesh, the mesh independent test is carried out through the change of mass flow and velocity at air outlet, as shown in Fig 6. The results show that when the mesh reaches 1.81 million, the change trend of these two parameters is relatively gentle. In order to reduce the calculation time while ensuring the calculation accuracy, a mesh containing 1.81 million is employed for subsequent simulation calculations.

## 3. Results and discussion

### 3.1 Airflow distribution in isolation ward

**Fig 7:**
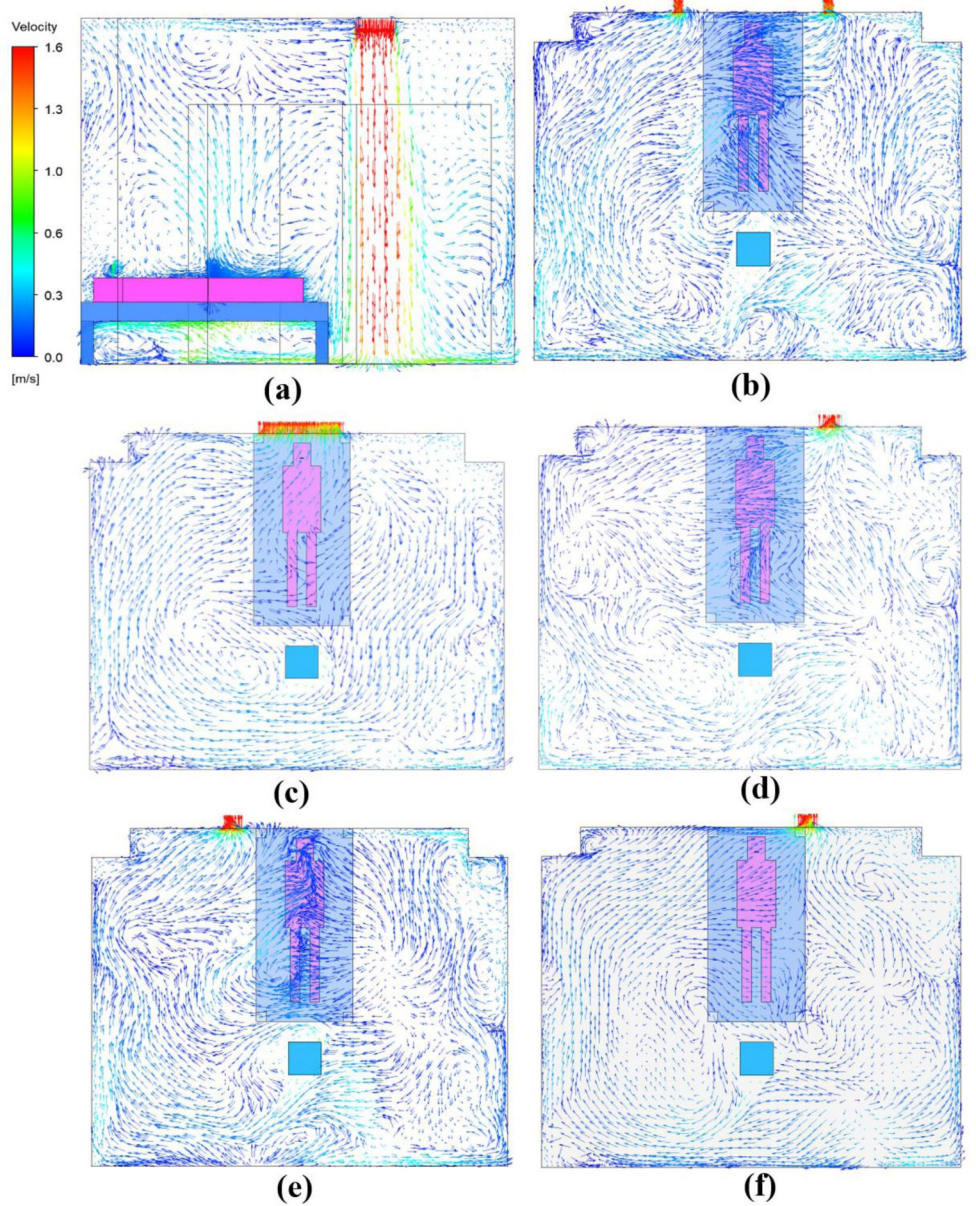
Airflow distribution pattern with the patient in the supine position. (a) Case1-sup (central plane); (b)Case1-sup(y = 800mm); (c)Case2-sup(y = 1700mm); (d)Case3-sup (y = 800mm); (e)Case4-sup (y = 800mm); (f)Case5-sup(y = 1400mm).

**Fig 8:**
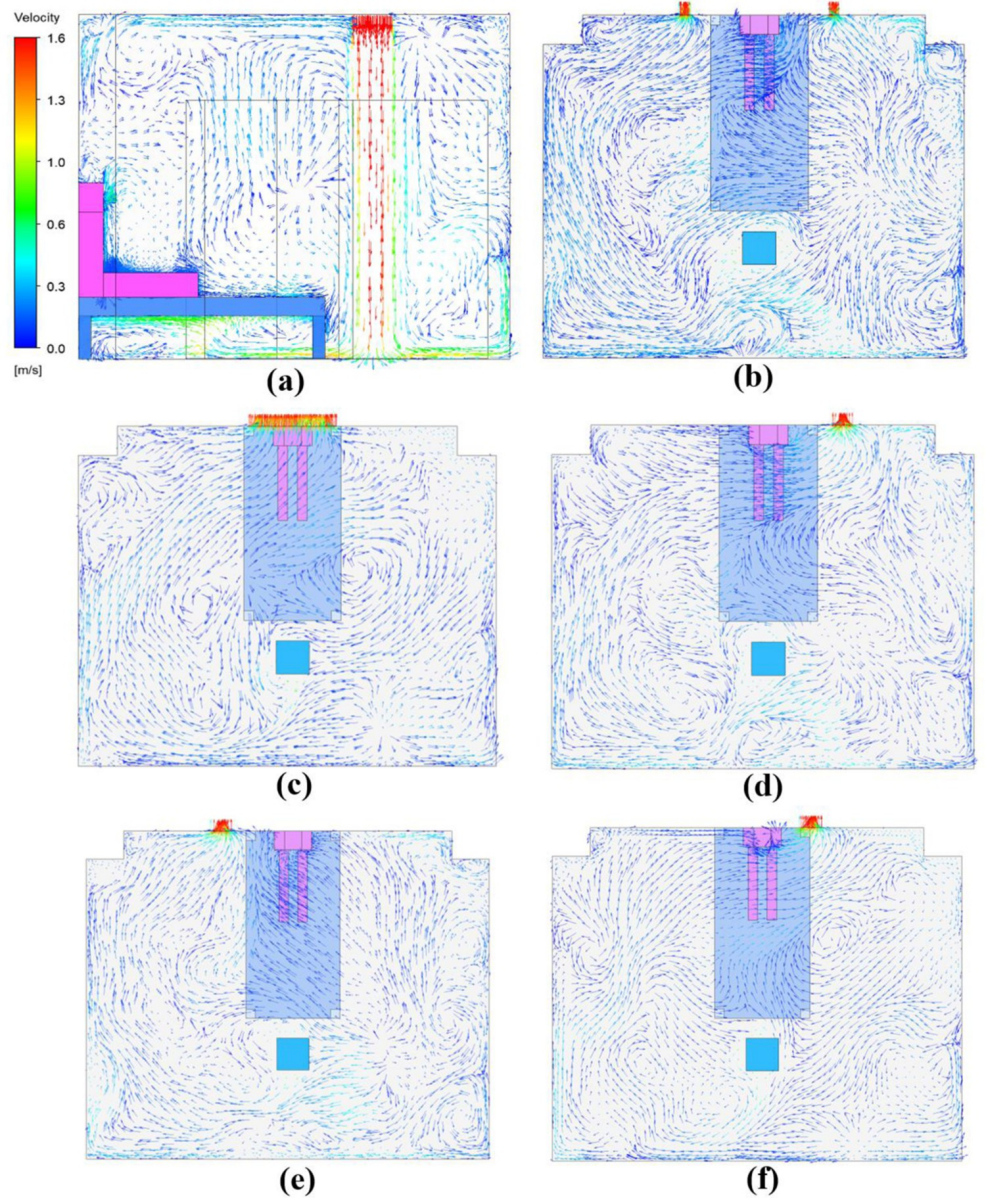
Airflow distribution pattern with the patient in the sitting position. (a) Case1-sit (central plane); (b) Case1-sit (y = 800mm); (c) Case2-sit (y = 1700mm); (d) Case3-sit (y = 800mm); (e) Case4-sit (y = 800mm); (f) Case5-sit (y = 1400mm).

The migration characteristic of droplet aerosols in the ward is strongly dependent on the airflow pattern. Fig 7 shows the airflow vector distribution at the center surface in case 1 (Fig 7(A)) and at the horizontal plane of exhaust air outlet in all five cases (Fig 5B–5F) when the patient is in the supine position. The air extends downward after leaving the air supply inlet before it diffuses sufficiently in the isolation ward to mix the airflow uniformly (Fig 7(A)). Some air forms a vortex field on the floor and above the patient, and similar phenomena occur in the rest of cases. In Case 1, most of the air flows to the right side of the exhaust due to the door gap air intake, resulting in an overall rightward airflow above the patient (Fig 7(B)). Case 2 has the exhaust outlet above the patient, bringing a higher velocity that facilitates contaminant removal (Fig 7(C)). For the rest of cases, because of the high negative pressure at exhaust outlet dominates, which in turn directs the air to the side of outlet (Fig 7D–7F). The airflow distribution pattern when the patient is in the sitting position is shown in Fig 8. After hitting the floor, the incoming air is dispersed in all directions, with some of it flowing vertically up the patient and being carried back through the ceiling into the supply airflow (Fig 8(A)). In the remaining cases (Fig 8D–8F), the airflow the airflow is found to form small swirling currents near the patient. When the exhaust setup of Case 2 is used (Fig 8(C)), the exhaled air from the patient can be discharged directly without causing recirculated air back into supply airstream, facilitating the removal of droplet aerosols.

The airflow mixing in the mentioned cases is relatively uniform, indicating the advantage of downward air supply in diluting contaminants, which is similar to the results obtained in previous studies. Due to the airflow pattern and interference from objects (bed, patient’s body), air recirculation occurs in the ward. This phenomenon was also observed by Dao et al. and Liu et al. However, the ventilation layout in this study is different from their work, resulting in different locations of air recirculation.

### 3.2 Spatial and temporal distribution of aerosol particles in isolation ward

**Fig 9:**
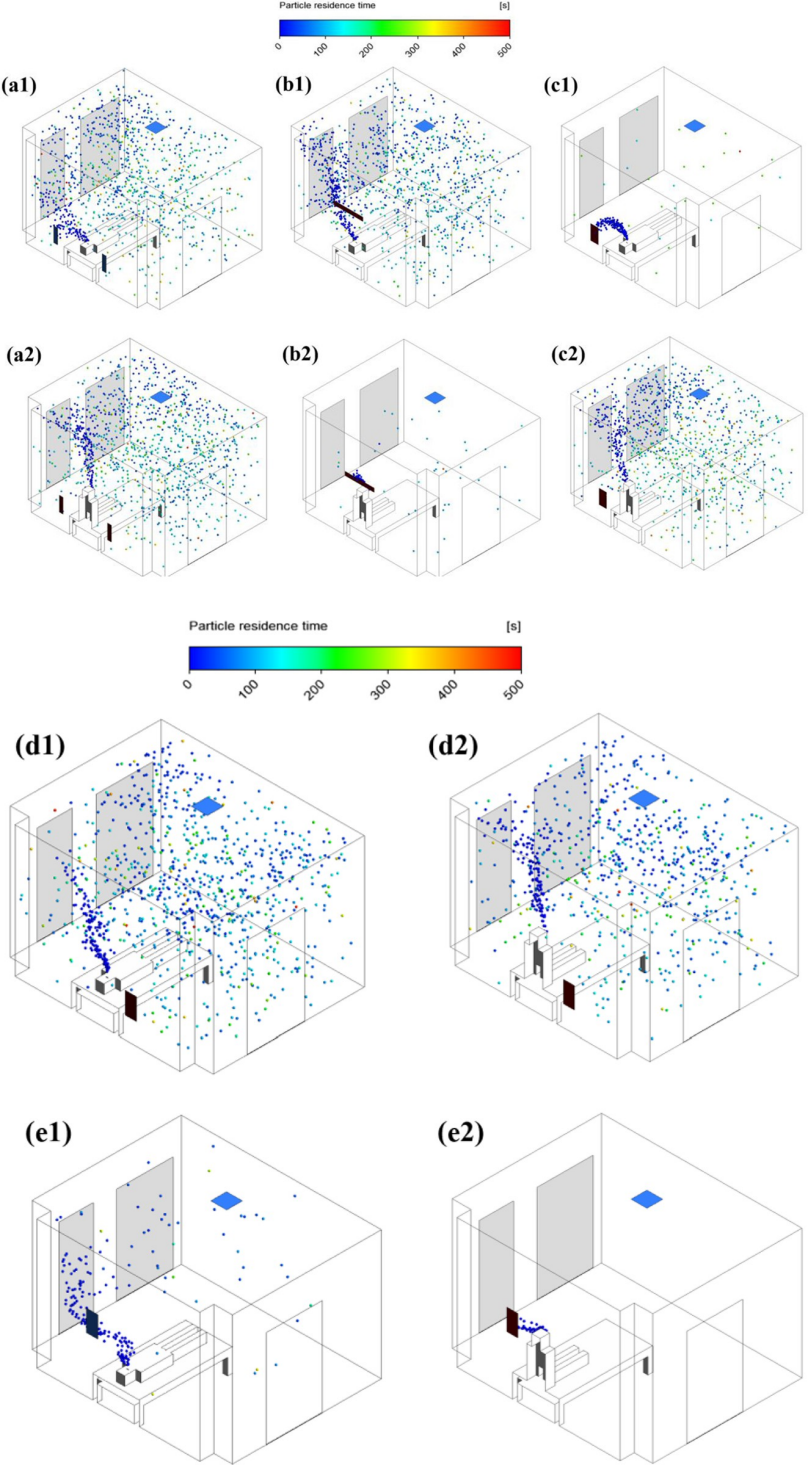
Spatial and temporal distribution of aerosol particles. (a1)Case1-sup; (a2)Case1-sit; (b1)Case2-sup; (b2)Case2-sit; (c1)Case3-sup; (c2)Case3-sit; (d1)Case4-sup; (d2)Case4-sit; (e1)Case5-sup; (e2)Case5-sit.

Fig 9 shows the spatial and temporal distribution of bioaerosol particles under different ventilation conditions. Among them, Fig 9A1–9E1 and 9A2–9E2 represent the particle distribution with the patient in supine and sitting positions, respectively. It can be found that both the ventilation mode and the patient’s body position have significant effects on the spatio-temporal distribution of particles. For the supine position, bioaerosol particles flow sideways in the initial stage, because the door gap air has a disturbance effect on the main airflow. For the sitting position, the particles move and diffuse upward after leaving the patient, basically in line with the airflow direction. It can be observed from cases 1–4 that the ventilation mode of case3 and case2 achieves the best particle removal efficiency in supine and sitting positions, respectively. In addition, under other cases, the particles are widely distributed in the isolation ward with significantly more residence time, which increases the risk of HCWs being infected, as displayed in Fig 9A1–9E1 and 9A2–9E2. Based on the effect of two patient postures on particle trajectories, we proposed an optimized ventilation model for case5 (Fig 9(E1) and 9(E2)). Compared with the above cases, the number of particles in case5 is smaller, and it has a good effect on the removal of exhaled particles when patients are in different positions.

In conclusion, the airflow field significantly affects the spatiotemporal distribution characteristics of particles, which is consistent with the results reported in previous literature. Similar to the work of Dao et al. [18], we also found that the particle removal efficiency is closely related to the position of the exhaust outlet. However, the difference lies in the optimal ventilation scheme derived. Dao et al. concluded that the best ventilation solution is to place the exhaust outlet above the patient’s head. In our study, considering the breathing scenario and the influence of patient positions, our optimal ventilation scheme differs from theirs.

### 3.3 Characteristics of deposition, removal and suspension of bioaerosols in isolation ward

**Fig 10:**
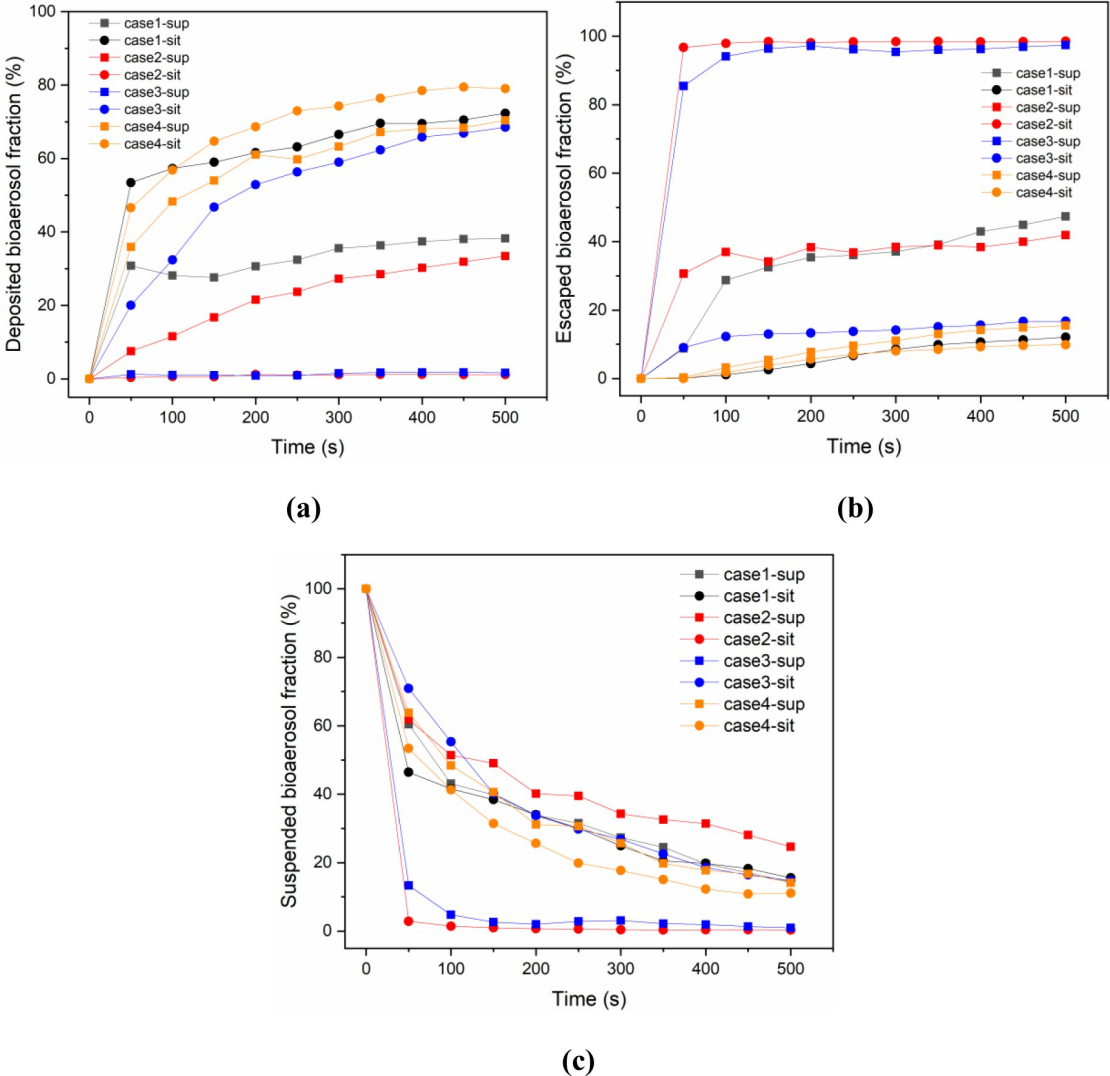
Migration of bioaerosols during 500s under different cases. (a) Deposited bioaerosol fraction; (b) Escaped bioaerosol fraction; (c) Suspended bioaerosol fraction.

Fig 10(A) gives the bioaerosol deposition at different cases during the simulated calculation of 500s. The deposited bioaerosol fraction is the ratio of the number of deposited particles on all surfaces to the total number of particles released. During the first 50 s, the deposition fraction increases rapidly for most scenarios, and then the trend slows down and stabilizes. This is because the distribution of particles in the room stabilizes over time under the mixing effect of airflow. Except for case2, the fraction of infector in the sitting position is higher than in the supine position. The reason for this phenomenon may be that changes in body position alter the distribution of airflow, which in turn affects the interaction of airflow and respiratory power dissipation. It can be seen that the deposited bioaerosol rate in case2-sit and case3-sup is almost 0, because most of the aerosol particles are removed. Fig 9B and 9C demonstrate the tendency of the bioaerosols escaping the ward through the exhaust outlet and suspending in the ward, respectively. As observed in Fig 10(B), the percentage of particles removed in case2-sit and case3-sit increases rapidly with time and reaches 98.55% and 97.42% at 500 s, respectively, indicating the good contaminant removal effect of both ventilation modes when the patients are in different body positions. However, the particle escape rates of case2-sup and case3-sit are only 41.91% and 16.74%. This means that the effect will be less satisfactory when the patient changes position. Compared with other cases, the particle removal rate of case4 is only 15.51% (supine position) and 9.86% (sitting position) at 500s, both of which are at a low level. The reason may be that this exhaust setup cannot form an effective directional airflow, causing a large number of particles to be deposited on various surfaces of the isolation ward or suspended in the air. Overall, the trend of bioaerosol suspension rate changes were basically consistent for all cases, as shown in Fig 10(C). As time passes, aerosol particles are gradually deposited and discharged from the exhaust air outlet, thus the difference between aerosol particle suspension rates at the same time point gradually decreases.

**Fig 11:**
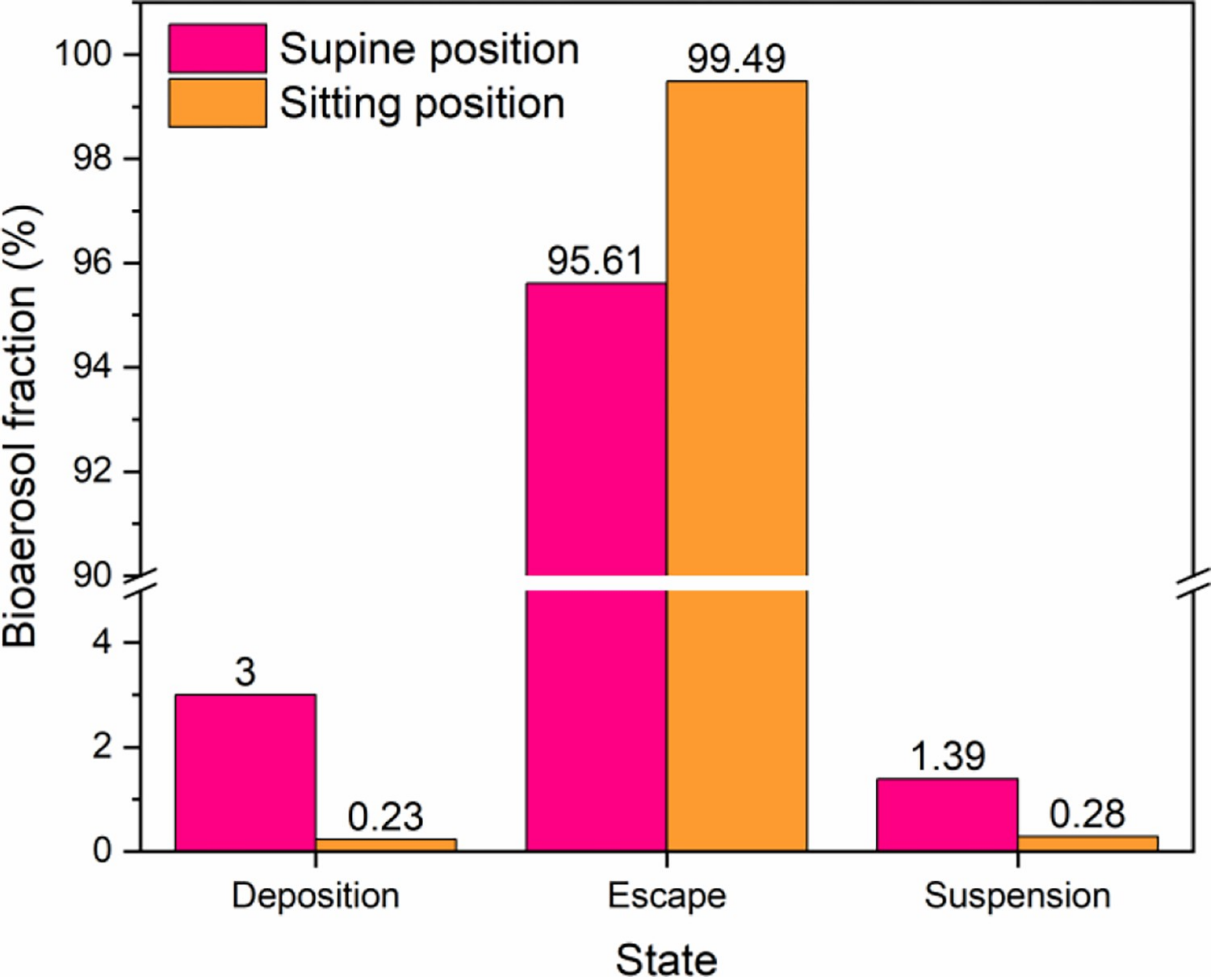
Migration of bioaerosols at 500s under the optimized scenario (case5).

The bioaerosol particles migration of the optimized scenario (case5) is presented in Fig 11. The results show that with the infector in the supine and sitting positions, the escaped particles exceed 95% of the total number of droplet particles, indicating that the ventilation efficiency is significantly improved after optimizing the ventilation pattern. Additionally, the lower fractions of bioaerosol deposition and suspension are able to reduce the infection probability of HCWs by direct contact transmission or airborne transmission.

**Fig 12:**
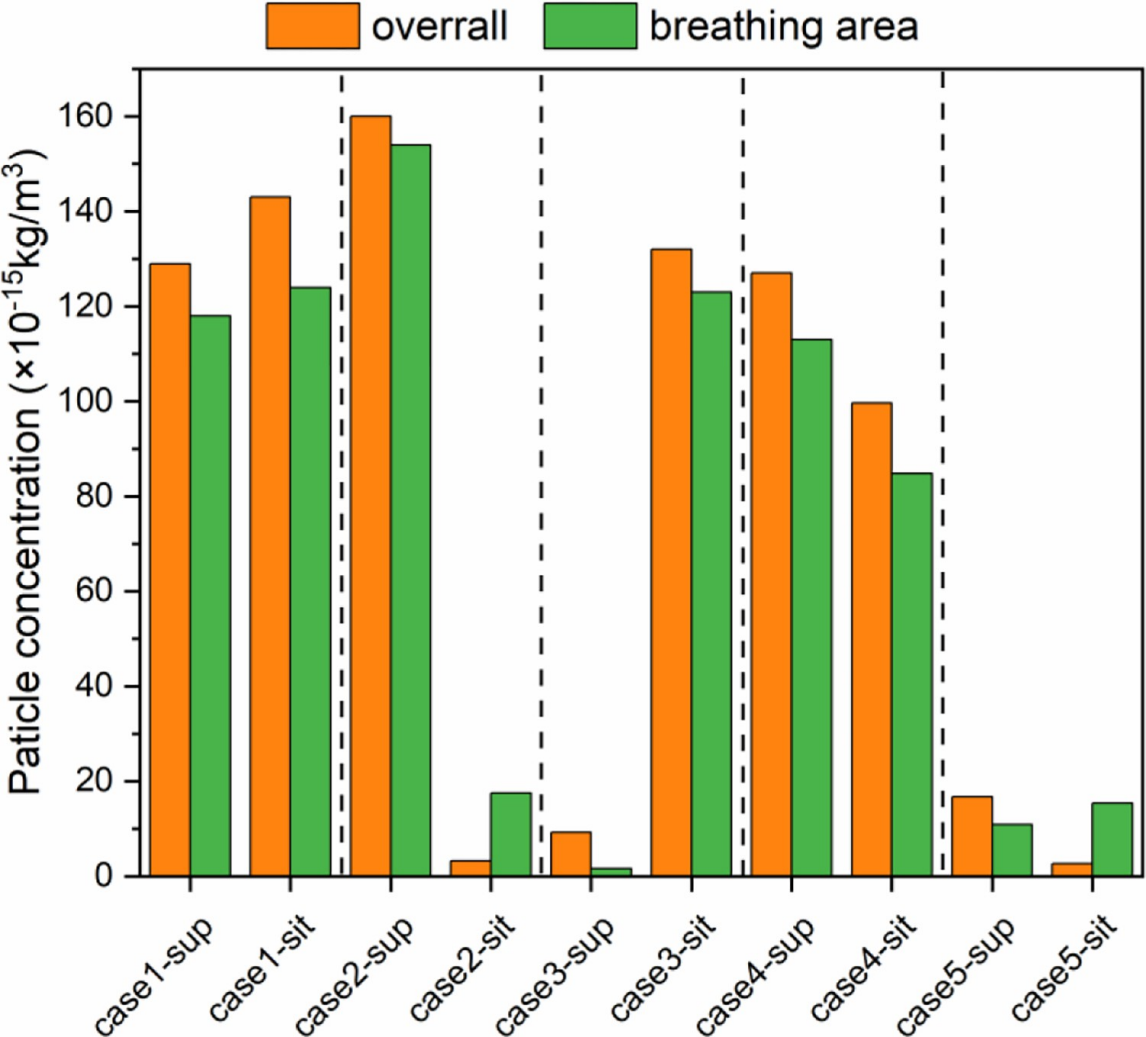
Particle concentration in the overall ward and breathing area of HCWs under different cases at 500s.

According to the results of Fig 10, the deposition, removal, and suspension curves of bioaerosols in each case have leveled off at 500 s, indicating that the bioaerosol distribution has reached a relatively stable state. Since the patient stays in the patient room for a long time, it is necessary to identify a time event that represents a relatively stable state for further research. Hence, the 500s time event is selected for particle concentration analysis. The particle concentrations in the overall ward and breathing area of HCWs under different cases at 500s are shown in Fig 12. HCWs have a large range of movement within the ward, but there is an approximate height range in their breathing zone. Thus, in accordance with Ref. [11], the space with the height of 1.3–1.7m is set as the breathing area of HCWs, which is the likely height for them to breathe. As for case1 and case4, the particle concentrations are in the range of 80×10^−15^–160×10^-15^kg/m^3^, but case4 performs better in removing pollutants. In case2, the particle concentration in the sitting position does not exceed 20 × 10^−15^ kg/m^3^, which is obviously lower than the concentration in the supine position. The reason is that when the patient is in the sitting position, his exhaled air will be directly exit, and there is no entrained return air back into the supply airstream. However, with the patient in the supine position, his breathing position is farther from the exhaust, and more bioaerosols are dispersed and suspended in the ward, resulting in higher particle concentrations. Obviously, case3 is the opposite of case2. In case3, the negative pressure zone formed by a single exhaust outlet is more concentrated, which, together with the air intake from the door gap, creates directional airflow in the supine area of the infector. This is why the particle concentration is lower in the supine position. The optimization results in case5, which can reduce the particle concentration to less than 20×10^-15^kg/m^3^ in all cases. This indicates that through ventilation mode optimization, directional airflow can be formed in areas where patients are in different body positions, reducing the residence time of particles in ward and improving ventilation performance.

**Fig 13:**
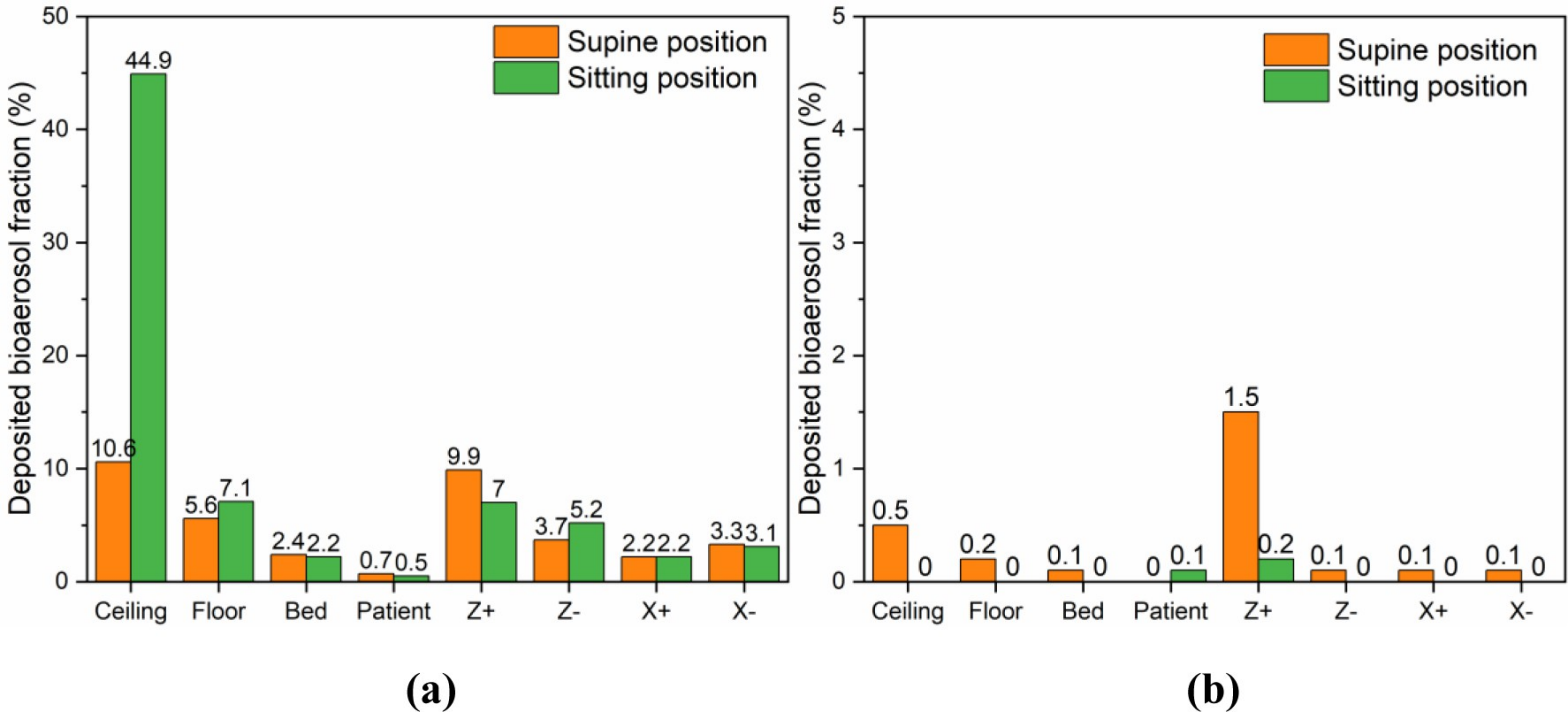
Different surface deposition fractions in the isolation ward under different cases. (a) Case1; (b) Case5.

Fig 13 reveals the deposition of bioaerosols on different surfaces of the ward within 500 s under the baseline case (case1) and the optimized case (case5). The deposited bioaerosol fraction is calculated as the number of particles deposited on different surfaces of the isolation ward as a percentage of the total number of particles released by the patient. The fraction of difference surfaces in the ward varies because of the location of the exhaust outlet. In both cases, more particles are deposited in the Z+ wall since the release source is closest to it, where the aerosol is subjected to the wrapping effect of the air stream. In case1, the highest deposition rate is found in the ceiling, with 10.6% and 44.9% respectively in the supine position and the sitting position. This is mainly due to the airflow pattern playing a dominant role in the particle migration. The upward flow near the release source entrains aerosol particles hitting the ceiling and then a large quantity of particles are deposited. Compared to the supine position, the sitting position has a higher location of the release source, which is less affected by the airflow near the exhaust outlet, and therefore more particles reach the ceiling. As for case5, there is a substantial reduction in the deposition rate on all surfaces. The optimization of ventilation pattern improves the removal of aerosol particles and also reduces their deposition rate.

Comparing a-b in Fig 13 reveals that the deposition fraction of patient body and bed in case5 are both 0.1%, which are significantly lower than in case1 (3.1% and 2.7%). As previously analyzed, improving the removal efficiency of bioaerosol particles by optimizing the ventilation pattern is the most critical measure. A well-directed airflow reduces the number and residence time of particles suspended in the ward, ultimately reducing the probability of particle deposition on patient and bed. Since HCWs must frequent contact with patients and bed surfaces, this can greatly decrease the risk of contact transmission, which helps to contain the spread of infectious diseases.

### 3.4 Limitation

1. The effect of the activities of HCWs on the flow field and particle distribution in the isolation ward does not be considered in this paper, which needs to be further researched.
2. Future research needs to systematically study the combined effects of breathing, coughing, speaking, and sneezing by patients.
3. Future research also needs to introduce infection risk prediction models to quantify the risk of cross-infection between HCWs and patients.

## 4. Conclusion

In this research, the transient simulation of a negative pressure isolation ward has been carried out to analyze the bioaerosol spatial distribution and migration characteristics, so as to evaluate the best arrangement of ventilation systems and rational strategies for contamination control. The simulation results reveal that the ventilation performance of ward can be improved by rearranging the location of exhaust air outlets when the patients are in different body positions. The ventilation layout of Case 5 is able to create a good directional airflow near the patient’s head, enhancing the ventilation efficiency. The ventilation layout of Case 5 is able to create a good directional airflow near the patient’s head, enhancing the ventilation efficiency. The bioaerosol removal efficiency in both the supine and sitting positions exceeded 95%, which is better than other cases. On this basis, the bioaerosol concentration in the ward of case 5 is greatly decreased (below 20 × 10^−15^ kg/m3), which reduces the probability of pathogens contracting HCWs through the airborne route. Instead of the concentration results, the proportion of aerosol particles deposited on all surfaces of the ward is also reduced, especially on the patient’s body and bed, which also helps to reduce the risk of cross-infection.
